# Using cognitive interviewing to bridge the intent‐interpretation gap for nutrition coverage survey questions in India

**DOI:** 10.1111/mcn.13248

**Published:** 2021-08-25

**Authors:** Sattvika Ashok, Sunny S. Kim, Rebecca A. Heidkamp, Melinda K. Munos, Purnima Menon, Rasmi Avula

**Affiliations:** ^1^ Poverty, Health, and Nutrition Division International Food Policy Research Institute New Delhi India; ^2^ International Health Johns Hopkins University Bloomberg School of Public Health Baltimore Maryland USA

**Keywords:** cognitive interview, India, intervention coverage, maternal and child nutrition

## Abstract

Designing survey questions that clearly and precisely communicate the question's intent and elicit responses based on the intended interpretation is critical but often undervalued. We used cognitive interviewing to qualitatively assess respondents' interpretation of and responses to questions pertaining to maternal and child nutrition intervention coverage. We conducted interviews to cognitively test 25 survey questions with mothers (*N* = 21) with children less than 1 year in Madhya Pradesh, India. Each question was followed by probes to capture information on four cognitive stages—comprehension, retrieval, judgement, and response. Data were analysed for common and unique patterns across the survey questions. We identified four types of cognitive challenges: (1) retention of multiple concepts in long questions: difficulty in comprehending and retaining questions with three or more key concepts; (2) temporal confusion: difficulty in conceptualizing recall periods such as “in the last 6 months” as compared to life stages such as pregnancy; (3) interpretation of concepts: mismatch of information being asked, meaning of certain terms and intervention scope; and (4) understanding of technical terms: difficulty in understanding commonly used technical words such as “breastfeeding” and “antenatal care” and requiring use of simple alternative language. Findings from this study will be useful for stakeholders involved in survey design and implementation, especially those conducting large‐scale household surveys to measure coverage of essential nutrition interventions.

**Key messages**

- Cognitive interviewing helps to identify problems in survey questions to improve question design and reduce measurement and response errors.
- Patterns of cognitive challenges in our study included retaining multiple concepts in long questions, temporal confusion pertaining to recall periods, interpretation of concepts, and understanding technical terms.
- Some solutions provided for improving survey questions may be applicable to multiple settings, but further cognitive interviewing may reveal context‐specific challenges useful for bridging the gap between intent and interpretation of questions.

## 1 INTRODUCTION

In many low‐ and middle‐income countries (LMIC), data about maternal and child health and nutrition intervention coverage are collected from large‐scale household surveys such as the Demographic and Health Surveys (DHS) and the Multiple Indicator Cluster Surveys (MICS). These surveys use structured questions to assess whether respondents and/or their family members received specific interventions. To elicit accurate responses, it is critical that questions clearly and precisely communicate the question's intent, even across diverse contexts and after translation into various languages.

Responding to questions involves an underlying psychological process with several interrelated tasks, which may be complicated by the type and design of the question (Schwarz, 2007). Survey questions used to measure coverage of maternal and child health and nutrition interventions normally include multiple components such as recall period, type of service provider and service provided. A typical questionnaire may ask about interventions received during different life stages. For some interventions, there is potentially a large time gap between when a service is received and when a survey is conducted, which could contribute to response errors. Conventional pretesting or pilot testing of survey instruments before data collection often does not formally address or document details of how respondents interpret questions, attribute meanings to terms and language used in questions, or retrieve memory attached to recall periods to answer questions (Presser et al., 2004).

Cognitive interviewing is an applied qualitative approach aimed at identifying problems in survey questions to improve question design and reduce measurement and response errors (Willis, 2017). Derived from social and cognitive psychology, cognitive interviewing helps to assess whether the respondent understands the question in the way it is intended by the researcher (Collins, 2003). Four cognitive stages are involved in answering a question: understanding the question (*comprehension*), recalling relevant facts (*retrieval*), making a judgement if needed (*judgement*), and giving the response (*response*) (Tourangeau, 1984). These stages function in a highly interconnected and non‐linear way. Through cognitive interviewing, the researcher elicits information from the respondent about each of these cognitive stages involved in answering a survey question (Beatty & Willis, 2007). This method does not validate the accuracy of response; rather, it improves the formulation of questions to reduce any discrepancies between question intent and respondent's interpretation. Cognitive interviewing is more time and resource intensive compared to conventional pretesting and, therefore, is often overlooked in survey development (Beatty & Willis, 2007).

Cognitive interviewing has been applied in LMIC to understand respondents' comprehension and recall of questions and to suggest question modifications in the Women's Empowerment in Agriculture Index (WEAI) in Bangladesh and Uganda (Malapit et al. 2017) and Haiti (Johnson, 2015). In another study that aimed to assess the appropriateness, acceptability, and comprehension of Likert scales (e.g., statements to measure the degree of opinion or attitude) related to micronutrient adherence in Ethiopia and Kenya, cognitive interviewing helped to identify question challenges but needed to be adapted according to cultural and education backgrounds of participants to reduce the burden of the process (Martin et al., 2017). More recently, this method was used to assess linguistic and cultural translations of a tool to measure respectful maternity care in India, which highlighted the value of cognitive interviewing in identifying question failures that would have otherwise not been detected in typical survey pilot testing. (Scott et al., 2019).

Every five or so years, the DHS and MICS programmes follow a rigorous process for revising their core questionnaires to include new indicators and corresponding questions. MICS has adopted cognitive interviewing primarily to examine the question comprehension process, while it has been used to a lesser extent by the DHS programme (Arnold & Khan, 2018).

This paper presents findings from cognitive interviews conducted with mothers in a rural district in Madhya Pradesh, India, about survey questions designed to measure coverage of maternal and child nutrition interventions. The study objectives were to elucidate how the questions intended for inclusion in large‐scale household surveys perform along the cognitive stages and to suggest modifications to improve these questions. We present the overall patterns of cognitive challenges, identify question elements that help to facilitate appropriate responses, and propose question changes where problems were identified, which may serve as the basis for cognitive testing in other settings or adaptation and use of similar survey questions.

## 2 METHODS

### 2.1 Study setting

This study was designed as part of formative research for a larger study to validate survey questions about counseling on infant and young child feeding received by mothers with children less than 1 year of age, that is, the accuracy of responses based on maternal recall. The validation study is under the Improving Measurement and Program Design (IMPROVE) project, funded by the Bill & Melinda Gates Foundation. An aim of the IMPROVE project is to improve quality and availability of maternal, neonatal, child and adolescent health and nutrition intervention coverage estimates generated by household surveys and other methods of data collection.

Our study was conducted in Madhya Pradesh, a large state in central India where overall coverage of health and nutrition interventions closely reflects the national averages (Bajaj et al., 2018). Hindi, the official language of India, is spoken in this region, which allowed for conducting cognitive interviews in one of the country's most common languages. One rural district was purposively selected based on physical access, common language and moderate health service coverage, and five villages were selected randomly as the study sites. Data collection lasted 1 week in October 2019.

In India, health and nutrition services to women and children are provided at public and private health facilities by doctors, nurses and health staff. At the community level, health and nutrition interventions are delivered by cadres of health and community workers, namely the Auxiliary Nurse Midwife (ANM), Accredited Social Health Activist (ASHA) and Anganwadi workers (AWW). ANMs are responsible for providing immunization, maternal and child health and family planning services, and health and nutrition education, while ASHAs promote immunization, give referrals for reproductive and other health services, and provide information on nutrition, sanitation and hygiene. AWWs deliver services targeted to pregnant and lactating women and children under 6 years such as supplementary nutrition, preschool non‐formal education, and nutrition and health education.

### 2.2 Study sample

The survey questions tested in our study pertain to nutrition interventions provided during pregnancy, delivery and early infancy; thus, mothers with children less than 1 year of age were eligible for the study. We estimated a sample size of 20 mothers to achieve saturation of ideas (Willis, 2005), separated in 2 subgroups: 10 mothers with children 0 to 5 months of age and 10 mothers with children 6 to 11 months of age. In each village, the AWW and/or her assistant provided a list of eligible mothers. Two mothers in each subgroup were randomly selected from the list, and those available at the time of the interview were invited to participate.

### 2.3 Survey questions tested

We tested 25 survey questions (See cognitive interview guide in the Supporting Information)—6 questions from the updated DHS‐8 core questionnaire published in October 2019 (The DHS Program, 2019), and 19 questions that were used in other household survey questionnaires such as the National Family Health Survey‐4, Alive & Thrive project and other evaluation surveys. Ten questions related to nutrition services during pregnancy (i.e., food or cash assistance, counseling on maternal nutrition, weight gain, iron, calcium supplements, and breastfeeding), 3 questions about services immediately after delivery (i.e., skin‐to‐skin contact and breastfeeding support), and 12 questions about services during early infancy (i.e., counseling about breastfeeding and complementary feeding). The questions on infancy will be used in the larger validation study. For the question about receipt of complementary feeding counseling, we included unprompted and prompted question formats to examine the difference in women's responses (Blair & Conrad, 2011).

### 2.4 Cognitive interviews

There are two common paradigms of cognitive interviewing ‐ think‐aloud technique and probing method. The think‐aloud technique involves the interviewer encouraging the respondent to verbalize his/her thoughts freely while answering. The probing method is directed by interviewer probes (Beatty & Willis, 2007). We chose the probing method for this study as interviewer‐directed probing is considered less burdensome for respondents compared to the open‐ended thinking‐aloud process (Collins, 2003). Each survey question was followed by probing questions to capture the four cognitive domains: comprehension, retrieval, judgement and response (Box 1).

**Table:** 

**Box 1: Examples of probing questions** **Comprehension:** *(In your own words) Can you tell me what I have just asked you?* *What do you understand by [TERM]?* **Retrieval:** *Who talked with you about [TOPIC]?* *How did you remember that [PERSON] talked to you about [TOPIC]?* *What did [PERSON] tell you about [TOPIC]?* **Judgement:** *Do you think other women would be hesitant or uncomfortable to answer this question? Why do you think so?* **Response:** *Was this question easy or difficult for you to answer? What did you find difficult?*

Interviews were conducted using a cognitive interview guide which included the 25 survey questions, each followed by 4–5 probing questions. Spontaneous or emergent probes were asked as needed (Beatty & Willis, 2007). The guide was translated into Hindi using the translation of similar questions and terms used in the National Family Health Survey‐4. The translated survey questions were pre‐tested for language accuracy, and the cognitive probes were pre‐tested to assess if they captured the full spectrum of issues faced by the respondents in interpreting and responding to the questions. Interviews were conducted in the respondent's home by a member of the research team (SA) accompanied by a note taker. Interviews were conducted by the first author who holds a master's level degree in social science with prior field research experience and proficiency in Hindi. The interviewer underwent orientation and training on the method, including through field visits to understand the study setting. The notetaker was proficient with the language local to the area and had prior experience in notetaking for qualitative research. Each interview lasted 45 min on average. Interviews were also audio recorded. Verbal (asking to repeat questions, side comments, etc.) and non‐verbal cues (long pause, frown, laughter, etc.) were captured in field notes by the note‐taker. Informed consent was obtained for all participants prior to starting the interview.

### 2.5 Data analysis

After each interview, field notes were entered into a matrix template in Excel. Audio recordings were used to complement field note entries. Responses in Hindi were simultaneously translated into English. Data were organized by each cognitive stage, then analysed for common and unique patterns and categorized into key findings. We also looked for patterns by child age subgroup and other sample characteristics (maternal age, education level, and birth order of the index child). Drawing on the typology of question failures (Scott et al., 2019) and mapping our data to previous literature (Hannan et al., 2020; Johnson, 2015), we used the cognitive stages as the basis of the analysis. In addition, facilitators or supportive question elements connected with better comprehension, retrieval and response were identified. While we did not focus our analysis on all variations in the Hindi translations or language, however, we did consider the translation issues and understanding regarding technical terms. If at least one‐quarter of respondents expressed similar difficulties in answering (parts of) questions, we also proposed specific question modifications.

### 2.6 Ethical considerations

Ethical approval was received from the Johns Hopkins Bloomberg School of Public Health Institutional Review Board (10010) and the International Food Policy Research Institute Institutional Review Board (00007490) in the U.S., and Suraksha Independent Ethics Committee in India. Approvals from the Madhya Pradesh Women and Child Development Department were also obtained. Verbal consent was obtained from all participants prior to interview.

## 3 RESULTS

### 3.1 Sample characteristics

A total of 21 interviews with mothers were conducted: 12 mothers with children less than 6 months of age and 9 mothers with children aged 6 to 11 months (Table 1). The mean age of respondent mothers was 24.4 years, and the mean age of index children was 5.7 months. Most mothers (85%) had completed primary or secondary levels of education. Most reported their occupation as housewives (67%) or farmers (29%). For more than half of the mothers, the index child was their second or later born child. We compared responses by maternal age (younger vs. older women), education level (primary school or lower vs. secondary school or higher) and index child birth order (first birth vs. later birth), and observed no differences in response patterns; thus, the following results are presented as overall patterns observed for the two subgroups defined by child age only.

**Table 1:** Sample characteristics

Characteristics	0–5 months *N* = 12	6–11 months *N* = 9	Total *N* = 21
	Mean/% (*N*)	Mean/% (*N*)	Mean/% (*N*)
Mean age of respondent mother (years, range)	26.0 (20–36)	22.2 (19–26)	24.4 (19–36)
Marital status: Married	100 (12)	100 (9)	100 (21)
Education level			
Never attended school	8.3 (1)	0	4.8 (1)
Primary school [Class 1–8]	41.7 (5)	55.6 (5)	47.6 (10)
Secondary school [Class 9–12]	41.7 (5)	44.4 (4)	42.9 (9)
University degree	8.3 (1)	0	4.8 (1)
Occupation			
Housewife	75 (9)	55.6 (5)	66.7 (14)
Farmer	16.7 (2)	44.4 (4)	28.6 (6)
Wage worker	8.3 (1)	0	4.8 (1)
Mean age of index child (months, range)	2.9 (0.4–5)	9.4 (6–11)	5.7 (0.4–11)
Birth order of index child			
First born	33.3 (4)	55.6 (5)	42.9 (9)
Second born	50 (6)	44.4 (4)	47.6 (10)
Third born or later	16.7 (2)	0	9.5 (2)
Sex of index child: Male	50.0 (6)	55.6 (5)	52.4 (11)

### 3.2 Cognitive challenges

We observed four main types of cognitive challenges—(1) retention of multiple elements in long questions, (2) temporal confusion, (3) interpretation of key concepts, and (4) understanding of technical terms. (Table 2). This typology is not mutually exclusive, and the challenges may be interrelated. The question‐specific results and suggested changes, where relevant, are presented in Tables 3a and 3b.

**Table 2:** Summary of cognitive challenges

Cognitive challenge	Explanation	Example
Retention of multiple elements in long questions	Difficulty in retaining 3 or more elements in long questions. Tendency to respond based on only the select elements retained.	“As part of your antenatal care during this pregnancy, did a health care provider do any of the following at least once …” contained at least 5 elements, and respondents had difficulty paraphrasing the entire question.
Temporal confusion	Uncertainty in the recall period. Questions referring to a specific life event/stage were less ambiguous compared to time interval/duration.	Questions referring to “during pregnancy” or “during delivery” were easier to recall compared to “in the last 6 months”.
Interpretation of key concepts		
(i) Mismatch of information being asked	Focus on select parts rather than the entire question leading to misinterpretation of intent. Questions on service exposure misconceived as questions about knowledge or practice, or questions taken out of context.	“What did the health care provider or community health worker talk with you regarding how or what to feed your child?” interpreted by respondent as “What do you know about child feeding?”
(ii) Different interpretation of the intervention	Varied interpretation on the range of activities/messages that constitute an intervention. Questions about receipt of counseling were considered differently depending on topic or brevity of discussion.	To the question about whether counseling about physical activity during pregnancy was received, a common response was “No, madam told me to take rest”; respondents did not consider being advised to rest or not lift heavy loads as advice about physical activity.
Understanding of technical terms	Plain and simple language to define (and translate) health and nutrition concepts is better understood than more technical terms.	“Feeding mother's milk” instead of “breastfeeding”, “check‐up during pregnancy” instead of “antenatal care”.

**Table 3a:** Summary of results for questions related to services during pregnancy and delivery

No.	Question	Response	Results by cognitive stage^a^	Proposed changes
Pregnancy (*N* = 21)				
1	During your pregnancy with [NAME OF YOUNGEST CHILD], did you ever receive food or cash assistance from government, an NGO, religious institution or other group?	Yes 47.6% (*N* = 10)	Difficulty in recollecting more than 2–3 elements (C), *N* = 10 Expressed need for question to be repeated (C), Poor understanding of food and cash assistance (C), *N* = 13 Clear recall of the last pregnancy period (R), *N* = 12 Expressed dejection by the delays in receiving assistance (J), *N* = 3	Shorten question to “… from the government or any other organization?” (only up to 2 entities or names of formal programmes). Consider separate questions for food assistance and cash assistance
2	What type of assistance did you receive?	Cash only 20% (*N* = 2); Food only 50% (*N* = 5); Both cash and food 30% (*N* = 3)		
3	As part of your antenatal care during this pregnancy, did a health care provider do any of the following at least once: A) Talk with you about which foods you should eat? B) Weigh you? C) Were you weighed at more than one antenatal care visit? D) Talk with you about your weight? E) Talk with you about being physically active?	A) Yes 81% (*N* = 17) B) Yes 100% (*N* = 21) C) Yes 95.2% (*N* = 20) D) Yes 52.4% (*N* = 11) E) Yes 76.2% (*N* = 16)	Identified specific health care providers (C), *N* = 12 Named family members as health care providers (C), *N* = 3 Poor understanding of “at least once” (C), *N* = 21 Recall of pregnancy period in general, but not antenatal visit specifically (R), *N* = 11 *Related to weighing, weight and physical activity:* Considered counseling/advice on weight as only when addressing a problem (C), *N* = 3 Did not consider advice about rest or reducing heavy workload as part of “physical activity” (C), *N* = 4 *‐* Clear recall of being weighed (i.e., when, where, by whom) (R), *N* = 18	Remove “at least once” Consider adding “physically active or taking adequate rest” based on country context
4	During your pregnancy with [NAME OF YOUNGEST CHILD], did a health care provider or community health worker talk with you about taking iron tablets or iron syrup?	Yes 90.5% (*N* = 19)	Poor understanding of general terms “health care provider” and “community health worker” (C), *N* = 11 Varied interpretation of what “talk with you” includes (C), *N* = 5 Good recall of information received about iron tablets, regarding its importance for their health and their baby's health (R), *N* = 14	Change “community health worker” to just “community worker” where community workers do more than health services Use common/local names of health care provider or community worker, as relevant [*for all questions containing “health care provider or community health worker”]
5	During your pregnancy with [NAME OF YOUNGEST CHILD], did a health care provider or community health worker talk with you about taking calcium tablets?	Yes 71.4% (*N* = 15) No 20.1% (*N* = 4) Do not know 9.5% (*N* = 2)	Did not know what calcium tablets are (C), *N* = 2 Good recall of information received about taking calcium supplements (e.g., to prevent weakness, for strength, etc.) (R), *N* = 13	None
6	When you were pregnant with [NAME OF YOUNGEST CHILD], did you receive any counseling about breastfeeding from any health care provider or community health worker?	Yes 76.2% (*N* = 16)	Translation used for “counseling” was “advice”, which was commonly used and well understood (C), *N* = 10 Misinterpreted health care providers or community health workers as family members (C), *N* = 4 Difficulty in understanding the technical term for “breastfeeding”, but easily responded when the terms “feeding mother's milk” used (C), *N* = 21 Recalled timeframe not limited to only pregnancy (R), *N* = 11	Use simple language (e.g., feeding mother's milk) for technical terms (“breastfeeding”), as relevant
Delivery (*N* = 21)				
7	Immediately after birth, was [NAME OF YOUNGEST CHILD] put on your chest?	Yes 61.9% (N = 13) No 33.3% (N = 7) Do not know 4.8% (N = 1)	Ease in paraphrasing all elements of the question (C), *N* = 19 Understood “immediately after birth” as within few minutes of giving birth (R), *N* = 17 Recall depended on their condition after delivery (J), *N* = 3 ‐ Response depended on physical condition immediately after birth (e.g., unconscious, in the ICU, child in the ICU, etc.) (A), *N* = 5	None
8	During the first two days after [NAME OF YOUNGEST CHILD]’s birth, did any health care provider or community health worker do the following: A) Talk with you about breastfeeding? B) Observe [NAME OF YOUNGEST CHILD] breastfeeding?	A) Yes 66.7% (N = 14) B) Yes 71.4% (N = 15)	Difficulty in recalling “first two days after birth” (C), *N* = 3 Asked to repeat the question, specifically the recall period (A), Interpreted “observe” as simply being seen (C), *N* = 3 Misinterpreted health care providers or community health workers as including family members (C), *N* = 5 ‐ Associated the time after delivery with the hospital stay (R), *N* = 14	Elaborate in 8B: “Observe you to check whether you are breastfeeding [CHILD] correctly”

^a^ Four cognitive stages are indicated as C—comprehension, R—retrieval, J—judgement, A—answer/response.

**Table 3b:** Summary of results for questions related to services for infants and young children

No.	Question	Response			Results by cognitive stages	Proposed changes
Infant and young child (0–5 months: *N* = 12, 6–11 months: *N* = 9)^a^						
1	In the last 6 months, did any health care provider or community health worker talk with you about how or what to feed your child?	0–5 months: Yes 50% (*N* = 6) 6–11 months: Yes 88.8% (*N* = 8)			Difficulty in recollecting all elements of the question (C), *N* = 21 Understood “what to feed” but did not “how to feed” (C), *N* = 16 Interpreted feeding as only food and not breastfeeding (C), N = 10 ‐ Misinterpreted “in the last 6 months” as child age (e.g., before child is 6 months, after the child is 6 months, or 6 months before child's birth/during pregnancy) (R), *N* = 8	Reinforce aid to recall “in the last 6 months” during enumerators training
2	In the last 6 months, what did the health care provider or community health worker talk with you regarding how or what to feed your child?		0–5 months	6–11 months	‐ Misinterpreted “in the last 6 months” as child age (R), *N* = 2	None
		Breastfeeding	16.7% (*N* = 2)	22.2% (*N* = 2)		
		Not feeding water or other liquids before 6 months, other than breastmilk	16.7% (*N* = 2)	11.1% (*N* = 1)		
		Introducing food and liquids (other than breastmilk) when the baby reaches 6 months of age	41.7% (*N* = 5)	0		
		Giving a variety of foods	16.7% (*N* = 2)	55.6% (*N* = 5)		
		Giving animal source foods (eggs, milk, meat, fish)	0	0		
		How often to feed foods	0	0		
		Not feeding sugar‐sweetened beverages	0	0		
		Not feeding unhealthy foods (sugary, salty or fried foods)	8.3% (*N* = 1)	0		
		Other (handwashing, cleaning food)	0	11.1% (*N* = 1)		
		Do not know	25% (*N* = 3)	11.1% (*N* = 1)		
3	In the last 6 months, did any health care provider or community health worker talk with you about breastfeeding?	0–5 months: Yes 75% (*N* = 9) 6–11 months: Yes 66.7% (*N* = 6)			Understood all elements the question (C), *N* = 21 Recalled “in the last 6 months” with respect to child age (R), *N* = 3	None
4	In the last 6 months, did any health care provider or community health worker talk with you about giving your child liquids, semi‐solid, or solid foods, other than breastmilk?	0–5 months: Yes 33.3% (*N* = 4) 6–11 months Yes 77.8% (*N* = 6)			Difficulty in understanding “liquids, semi‐solid and solid foods” (C), *N* = 4 Defined semi‐solid foods as “soft foods” (C), *N* = 8 Expressed that other mothers will have difficulty understanding this question (J), *N* = 4	Replace with “soft or solid foods, or liquids other than breastmilk?” and reordering liquids after foods (before breastmilk) so it is not forgotten.
5	In the last 6 months, did a health care provider or community health worker talk with you about: A) Not feeding water or other liquids before 6 months, other than breastmilk B) Introducing food and liquids (other than breastmilk) when the baby reaches 6 months of age C) Giving a variety of foods D) Giving animal source foods (such as eggs, milk, meat or fish) E) Not feeding sugar‐sweetened beverages F) Not feeding sugary, salty or fried foods	A) 0–5 months: Yes 66.7% 6–11 months: Yes 50% B) 0–5 months: Yes 41.7% 6–11 months: Yes 100% C) 0–5 months: Yes 33.3% 6–11 months: Yes 62.5% D) 0–5 months: Yes 16.7% 6–11 months: Yes 37.5% E) 0–5 months: Yes 25% 6–11 months: Yes 25% F) 0–5 months: Yes 25% 6–11 months: Yes 25%			Difficulty in keeping track of 2 reference time periods (“in the last 6 months” in the stem question and “before 6 months” in 5A and 5B) (C), *N* = 6 Did not associate milk as an animal source food such as meat, fish and eggs. (C), *N* = 4 Not familiar with the term “sugar‐sweetened beverages”; most respondents did not mention sugar‐sweetened beverages available in the market (C), *N* = 12 Considered “sugary, salty or fried foods” as foods that are sold in the market and snacks made at home (C), *N* = 12,15 ‐ Considered “unhealthy foods” as those bought from the market, mostly fried (C), *N* = 12	Consider adding “milk and other animal source foods” to make milk more explicit in 5D Discuss local examples of 5E and 5F during enumerators training
6	In the last 6 months, where did the health care provider or community health worker talk with you about how or what to feed your child?		0–5 months	6–11 months	Question understood by all mothers (C), *N* = 20 Recalled moments of going/taking child to the Anganwadi center for other services such as immunization, growth monitoring, and take‐home rations (R), *N* = 13 All mothers named at least one place (A), *N* = 20	None
		At home	0	14.3% (*N* = 1)		
		At Anganwadi center	77.8% (*N* = 7)	71.4% (*N* = 5)		
		At health center	0	14.3% (*N* = 1)		
		Hospital	44.4% (*N* = 4)	28.6% (*N* = 2)		
		Private clinic	0	0		
		Outreach facility	0	0		
		NGO facility	0	0		
		In the community/village	0	0		
		Other	11.1% (*N* = 1)	14.3% (*N* = 1)		
7	In the last 6 months, which health worker talked with you about how or what to feed your child?		0–5 months	6–11 months	Question understood by most mothers (C), *N* = 13 Identified the Anganwadi worker most often as having talked with them about child feeding, given highly frequent contacts with them in their communities (R), *N* = 14 All mothers named at least one service provider (A), *N* = 16	None
		Anganwadi worker	77.8% (*N* = 7)	100% (*N* = 7)		
		ASHA	44.2% (*N* = 4)	14.3% (*N* = 1)		
		ANM	11.1% (*N* = 1)	0		
		Doctor	0	28.6% (*N* = 2)		
		Pharmacist	0	0		
		NGO worker	0	0		
		Other (family, AWH, nurse)	11.1% (*N* = 1)	28.6% (*N* = 2)		
		Do not know	0	0		

^a^ For Q4–5, 0–5 months: *N* = 12 and 6–11 months: *N* = 8; for Q6–7, 0–5 months: *N* = 9 and 6–11 months: *N* = 7.

### 3.3 Retention of multiple elements in long questions

Questions containing three or more elements (e.g., more than one phrase to describe the recall period, type of intervention, and service provider type) posed a challenge in retention. Mothers did not grasp all elements of the question at first and asked for such questions to be repeated. When asked to paraphrase the question back to the interviewer, mothers tended to miss one or more of the question elements which altered its meaning. On probing, we observed how the missed elements of the question contributed to their interpretation and subsequent response.

For example, the question “During your pregnancy with [CHILD], did you ever receive food or cash assistance from government, an NGO, religious institution or other group?” contains seven different elements. When this question was first asked, many mothers paused and asked the question to be repeated (Table 3a, Q1). In paraphrasing, several mothers named either food or cash and/or only one or two sources of assistance. Some mothers responded that they received food and money from family, friends, or neighbors, indicating that they were answering based on select question elements retained. To shorten the question, we propose to include only up to two main entities or names of formal programmes that offer such assistance. Additionally, the question may be split into two separate questions asking about food assistance and cash assistance.

In the question “As part of your antenatal care during this pregnancy, did a health care provider do any of the following at least once …? ”, mothers omitted or did not understand “at least once” in the context of the question (Table 3a **, Q3**). We observed that “at least once” was a redundant concept. To make the question more concise, we propose to remove the phrase “at least once.”

### 3.4 Temporal confusion

Temporal confusion relates to the uncertainty in retrieving correct memory attached or anchored to a recall period. Survey questions tested in this study included recall periods of “during pregnancy”, “as part of antenatal care”, “immediately after birth”, “during the first two days after birth” and “in the last 6 months”. Recall periods referring to a time interval or duration (e.g., in the last 6 months) rather than a life stage (e.g., pregnancy) were more difficult for respondents to comprehend and retrieve from memory. Life events or stages provided better anchors to memory than intervals in time units. Some mothers asked interviewers to repeat the recall period in the questions before responding, indicating potential difficulty in memory retrieval.

For “immediately after birth”, most respondents correctly described it as within few minutes of giving birth (Table 3a, Q7). However, some mothers who said they were unconscious or not lucid after delivery still responded to the question based on information received from family or others present during delivery, which may be prone to error.

More than half of the mothers recalled “in the last 6 months” in association with their child's age, which led to various interpretations (Table 3b, Q1–Q7). One mother of a child aged 6 months explained, “I was thinking about the time since immunization when my child was three and a half months old.” For the question “In the last 6 months, did any health care provider or community health worker talk with you about how or what to feed your child?”, some women were confused by whether the question was referring to advice received about child feeding until or after 6 months of age, rather than correctly recalling the 6‐month period prior to the interview (Table 3b, Q1). We suggest providing guidance during enumerators' training on how to aid understanding of the 6 months recall period using relevant local event calendar or another context. This could also be enabled on computer‐assisted personal interviewing tools by automatically reframing the question to include the child's age.

### 3.5 Interpretation of key concepts

#### 3.5.1 Mismatch of information being asked

Regardless of question length or its complexity, some respondents focused on certain parts of the questions and misunderstood the overall meaning or intent. For the question “During the first two days after [CHILD]’s birth, did any health care provider … observe [CHILD] breastfeeding?”, mothers were able to paraphrase the entire question but focused on “observe breastfeeding” when responding (Table 3a, Q7B). A common response to this question was “yes, my family members/other women were around and saw me [breastfeeding].” Inasmuch as the synonyms of “observe” include watch, see, and notice, mothers responded “yes” to this question if anyone observed them breastfeeding, potentially resulting in overreporting on receipt of skilled breastfeeding support. We propose to rephrase this question to “Observe you to check whether you are breastfeeding [CHILD] correctly” to reduce ambiguity in the key concept of this question, that is, being observed to assess positioning and identify cues. Regardless of whether the mother was breastfeeding correctly or incorrectly at that time, we intend for this question on observed breastfeeding to be considered with an assessment of “correctness” or skillfulness in mind.

For the question about the child feeding information received during counseling, respondents misinterpreted “In the last 6 months, what did the health care provider … talk with you about regarding how or what to feed your child?” as a question about their child feeding knowledge/practice (Table 3b, Q2). Mothers focused on the “what to feed their child” and provided information on what they currently knew or how they practiced child feeding. Some mothers did not differentiate between the content of counseling received and their own pre‐existing or acquired knowledge of child feeding. We do not propose any changes to this question, although we recommend caution in the usage and interpretation of this question.

#### 3.5.2 Different interpretation of the intervention

Some terms such as “physical activity”, “weight gain” or “animal source foods” were misinterpreted unidirectionally or partially rather than wholly. For the question about whether counseling about physical activity during pregnancy was received, some mothers explained that they were told to take rest, not to lift heavy objects, reduce workload, etc. (Table 3a, Q3E). However, other mothers did not consider these advices as counseling on physical activity, which they understood as being about more exercise. Although the global recommendation on antenatal care is for “counseling about healthy eating and keeping physically active during pregnancy to stay healthy and to prevent excessive weight gain” (World Health Organization, 2016), women are often advised to rest and reduce workload in countries or contexts where underweight or inadequate weight gain during pregnancy persists (Ministry of Health and Family Welfare, n.d.). The term “physical activity” is defined as any bodily movement requiring energy expenditure, and counseling on this topic includes advice about doing more or less of such, as appropriate. To avoid any confusion, we propose to add the phrase “… about being physically active or taking adequate rest” to this question.

Regarding “...giving animal source food (such as eggs, milk, meat or fish)?”*,* some mothers did not associate milk as an animal source food (Table 3b, Q5D). Milk is commonly consumed in vegetarian diets, thus not always perceived as an animal source food. We propose to add “milk and other animal source foods (such as eggs, meat or fish)”, so that milk is made explicit in the question.

### 3.6 Understanding of technical terms

This cognitive challenge relates to language and translation as well as word choice. Technical terms pose a common challenge. Although the Hindi translation for terms such as “antenatal care” and “breastfeeding” are standardized and widely used in print or media, by service providers, etc., respondents may be familiar with more local terms that use plain and simple language to describe these concepts. When the common Hindi term for “antenatal care” was used, a few mothers did not understand initially. Mothers explained that the term meaning “checkup during pregnancy” was more familiar to them (Table 3a, Q3). Similarly, for “breastfeeding”, mothers understood the term when it was translated as “feeding mother's milk” (Table 3a, Q6). Some respondents were not familiar with the general terms “health care provider” or “community health worker”, until local or plain names or titles were used (Table 3a, Q4). Furthermore, community workers provide more than just health and nutrition services in many contexts, so we propose to change “community health worker” to “community worker”, as relevant.

### 3.7 Cognitive facilitators

#### 3.7.1 Prompted question format

Nearly all the survey questions tested in our study had binary response options. For the question on counseling about child feeding, however, we tested both unprompted and prompted versions to examine the cognitive process involved to respond. In the unprompted version of the question “In the last 6 months, what did the health care provider … talk with you about regarding how or what to feed your child?”, respondents were allowed to answer openly (Table 3b, Q2). Their responses were coded by the interviewer to any of the 8 response options based on key child feeding messages, none of the above, other, or do not know. The prompted version asked explicitly about each of the 8 key child feeding messages (Table 3b, Q5). As anticipated, more mothers responded positively to the prompted questions, compared to the unprompted question. The unprompted question captured the most salient responses but not the most comprehensive. While the prompted questions may have facilitated more comprehensive responses, the responses are not necessarily more accurate and may also result in overreporting. Still, the prompted question format reduced the cognitive burden on respondents.

#### 3.7.2 Questions about product‐oriented services or physical measurement

Survey questions on intervention coverage included both information‐oriented counseling services and more tangible product‐oriented services such as distribution of iron and folic acid supplements and weight gain monitoring (Kosec et al., 2015). Mothers could recall the time, location and service provider more quickly and responded more easily to questions attached to tangible interventions compared to those involving information sharing, corroborating similar findings from previous validation studies on antenatal care and postnatal care interventions (McCarthy et al., 2020).

#### 3.7.3 Short recall interval

The time interval between service receipt and survey interview is a critical factor influencing recall. Mothers in our study were up to 12 months postpartum at the time of interview and were asked about services received during their last pregnancy and ongoing services for their infants and young children. In general, mothers reported that the time interval was adequate to recall the services in question. Regarding questions about services received during her last pregnancy, one mother of a one‐month‐old child remarked, “It has not been a long time now. I may have forgotten if it was longer, busy in raising my child”. The relatively short interval between the most recent pregnancy and the interview facilitated the recall of information.

## 4 DISCUSSION

Our study identified key cognitive challenges faced by respondents in answering survey questions on maternal and child nutrition intervention coverage, many of which have been used in previous large‐scale surveys. The study also highlights the importance and utility of cognitive interviewing in developing or modifying survey questions that accurately communicate question intent and facilitate appropriate responses, as even assumed straightforward questions about whether a mother received counseling about child feeding may not be understood as intended. Our findings of cognitive challenges –retention of multiple elements in long questions, temporal confusion, interpretation of concepts, and understanding of technical terms – and of question elements that help to relieve cognitive burden are likely relevant in other contexts and for survey questions related to other topics, as corroborated by lessons from other cognitive interviews.

It was unsurprising that lengthy questions containing multiple elements led to poor comprehension and retention in memory. Long questions made it difficult for respondents to keep track of the intent of the question and thereby miss its core element (Scott et al., 2019). If it is not possible to simplify the question, enumerators may need to read such questions more than once and be prepared to offer an explanation where required.

Given that maternal and child nutrition interventions are delivered at different life stages or frequencies (e.g., during pregnancy, at delivery, every few months during early childhood, etc.), various recall periods will inevitably be used in survey questions about coverage of these interventions. We observed that recall periods referring to a life stage such as pregnancy rather than time intervals or duration were more easily understood and retrieved from memory by respondents. However, another study found that maternal recall of interventions that take place during and immediately after labor and delivery have low levels of validity, especially those concerning timing or sequence of activity (McCarthy et al., 2016; Stanton et al., 2013). Overall, life events or stages could still provide better anchors to experiential memory than intervals in generic time units. Other studies similarly identified that specific time periods led to better recall compared to broad and general time intervals (Choufani et al., 2020; Wilson, 2002), and questions on health‐related resource use “in the last 6 months” required significant probing to arrive at a response (Chernyak et al., 2012).

Concepts linked to the information asked, meaning of certain terms or intervention scope were subject to misinterpretation often due to respondents' focus on a select part(s) of questions or confusion between intervention received and their previous knowledge/practice. Where the meaning of terms or interventions are prone to narrowed or misinterpretation, as presented in the examples of “physical activity” or “animal source foods” in our study, clarification or examples should be readily on hand. A study on respectful maternity care noted that alternative explanations should be ready and provided where respondents understand different words to refer to a specific concept, for example, to “squat” in position for delivery also explained as “knee bent or sitting on toilet position” (Scott et al., 2019). Several studies have highlighted errors pertaining to survey items not measuring constructs they intended to measure, variable interpretation of terms, misinterpretation or limited understanding of terms stemming from different cultural contexts, etc. (Carbone et al., 2002; Levine et al., 2005; Schildmann et al., 2016). To reduce ambiguity in these cases, researchers have revised or clarified the wording in question, especially in contexts where culturally relevant adaptations of standardized questionnaires have been done. Similarly, we proposed to add clarifying words within questions where relevant.

Difficulties in comprehension of technical terms by the general population are widely acknowledged, thus require careful consideration of language and word choice. Several studies observed that use of technical jargon reduced the scope of understanding of the terms intended (Johnson, 2015) and posed both wording and conceptual difficulties in measuring constructs (e.g., quality of life) that are not relatable to specific population groups (Zeldenryk et al., 2013). To address such issues, cognitive interviewing has also been used to improve health surveys, such as those on illness perception, by adapting them to be culturally relevant (Shiyanbola et al., 2019).

For our study, the sample of respondents included only mothers with children up to 1 year of age. We focused on this age group because our study mothers were more likely than mothers with older children to have been targeted for the full range of interventions from pregnancy to early childhood, and, therefore, could provide responses to all questions during the cognitive interviews. Additionally, the cognitive interviews were embedded in a larger validation study targeted to this age group. In many large‐scale surveys, questions related to maternal and child intervention coverage will likely address children older than one year of age and over longer recall periods (e.g., 3–5 years since last pregnancy) (The DHS Program, n.d.). However, we consider our study findings to be relevant for survey questions for mothers with children beyond the first year of age. Another limitation is that we conducted a single round of cognitive interviews, compared to multiple iterations as was done in some studies. However, through the single round of in‐depth interviews that included alternate versions of the question, we were able to obtain rich findings about the cognitive processes and more generalizable ways to improve questions. For use of questions in specific contexts, we recommend additional rounds of testing and modification as relevant.

## 5 CONCLUSION

Cognitive interviewing helps to preempt question failures by identifying the cognitive challenges, thereby reducing measurement errors. While some solutions for improving survey questions may be applicable to multiple settings, cognitive interviews identify context‐specific challenges useful for bridging the gap between intent and interpretation of questions. Our study results present a case on ways to revise and improve questions about nutrition intervention coverage. To address the question of response accuracy specifically related to nutrition counseling, a validation study applying some of the survey questions tested through cognitive interviews is planned as a next step. We recommend that cognitive interviews be applied especially when new questions are being added to surveys. The findings from our study may be particularly useful as the basis for other cognitive testing in varied settings and for stakeholders involved in the design and implementation of large‐scale household surveys, aiming to capture and improve coverage data on maternal and child nutrition interventions.

## CONFLICT OF INTEREST

The authors declare that they have no conflicts of interest.

## CONTRIBUTIONS

SSK and RA designed the research study; SA drafted and revised the manuscript and conducted data collection; SA, SSK and RA analysed the data; RAH, MKM and PM provided inputs and reviewed the manuscript.

## Supporting information


**Table S1.** MICYN COUNSELING QUESTIONS

## Data Availability

The data that support the findings of this study are available from the corresponding author upon reasonable request.
