# Supplementary material for: Using cognitive interviewing to bridge the intent‐interpretation gap for nutrition coverage survey questions in India
Source: Matern Child Nutr. 2021 Aug 25;18(1):e13248. doi: 10.1111/mcn.13248 (PMC8710093; doi:10.1111/mcn.13248)
Supplement: Supplementary file 1 — Table S1. MICYN COUNSELING QUESTIONS [file MCN-18-e13248-s001.docx]

#

# MODULE 3. MICYN COUNSELING QUESTIONS

| **NO.** | **QUESTION** | **RESPONSE** |
| --- | --- | --- |
| **NUTRITION INTERVENTIONS DURING PREGNANCY/ANTENATAL CARE** | | |
| **301** | During your pregnancy with [*NAME OF YOUNGEST CHILD*], did you ever receive food or cash assistance from government, an NGO, religious institution or other group?  **जब (बच्चे क नाम) पेट में था, क्या आप को कभी सरकार, एक गैर सरकारी संगठन, धार्मिक संस्था या अन्य समूह से भोजन या पैसों की सहायता मिली थी?** | No नहीं 0  Yes हां 1  Does not know पता नहीं.............................................................98 |
| **302** | What type of assistance did you receive?  **आपको किस तरह की सहायता मिली थी?** | Cash only सिर्फ पैसे ...1  Food only सिर्फ भोजन 2  Cash and food mix दोनों, भोजन और पैसे 3  Other (specify: _____) अन्य (बताएं...) 95 |
| a | (In your own words) Can you tell me what I have just asked you?  क्या आप मुझे बता सकते हैं कि मैंने अभी आपसे क्या पूछा? |  |
| b | What do you understand by food assistance?  भोजन सहायता से आप क्या समझते हैं? |  |
| c | What do you understand by cash assistance?  पैसे की सहायता से आप क्या समझते हैं? |  |
| d | Which time were you thinking of while answering this question?  इन सवाल के जवाब देते समय आप किस वक्त के बारे में सोच रहे थे? |  |
| e | Was answering this question easy/ difficult for you?  क्या आप को इस सवाल का जवाब देना आसान लगा की मुश्किल लगा? |  |
| f | Why did you feel so?  आपको ऐसा क्यों लगा? |  |
| g | Do you think other women would be hesitant or uncomfortable to answer this question?  क्या आपको लगता है की आपकी जैसे दूसरी महिलाओं को इस सवाल के जवाब देने में हिचकिचाहट होगा या डर लगेगा? |  |
| h | If yes, why do you think so?  यदि हाँ, तो आप को ऐसा क्यों लगता है? |  |
|  | **Miscellaneous/ Additional Probes:** |  |
| **303** | As part of your antenatal care during this pregnancy, were any of the following done at least once:  **इस गर्भावस्था की जांच के दौरान क्या कम से कम एक बार** |  |
|  | 1. Did a health care provider talk with you about which foods to eat while pregnant?   **क्या कोई स्वास्थ्य प्रदानकर्ता ने गर्भावस्था के दौरान क्या खाना चाहिए - इसके बारे में आपसे बात किया?** | No नहीं 0  Yes हां 1  Does not know पता नहीं 98 |
| a | What does at least once mean to you?  कम से कम एक बार का मतलब क्या हो सकता ह? |  |
| b | What do you understand by antenatal care? (checkup before delivery)  गर्भावस्था की जांच से आप क्या समझते हैं? |  |
| c | When I said, healthcare provider, who did you think of while responding?  जब मैंने स्वास्थ्य प्रदानकर्ता कहा तो आपने किन के बारे में सोच कर जवाब दिया? |  |
| d | Sometimes, it is difficult to remember what happened during pregnancy, how did you remember you were told about which foods to eat while pregnant?  आम तौर पर गर्भावस्था के दौरान हुई बातों को याद रखना मुश्किल होता है\| आपको ये कैसे याद है कि गर्भावस्था के दौरान आपको खाने के बारे में बताया गया था? |  |
| e | What were you told about what to eat while pregnant?  गर्भावस्था के दौरान के खाने के बारे में क्या बताया गया था? |  |
| f | Was this question easy or difficult for you to answer?  क्या आप को इस सवाल का जवाब देना आसान लगा की मुश्किल लगा? |  |
| g | Why did you feel this way?  आपको ऐसा क्यों लगा? |  |
| h | Do you think other women like you will find be able to respond to these questions? Or will they find it difficult?  क्या आपको लगता है की आप जैसी दूसरी महिलाऍं इस प्रश्न का उत्तर दे पायेंगी? या उनको मुश्किल लगेगा? |  |
| i | Why do you think so?  यदि मुश्किल लगा हो तो, ऐसा क्यों लगा था? |  |
|  | **Miscellaneous/ Additional Probes:** |  |
| **303** | As part of your antenatal care during this pregnancy, were any of the following done at least once:  **इस गर्भावस्था की जांच के दौरान क्या कम से कम एक बार** |  |
|  | 1. Did a health care provider weigh you?   **क्या किसी स्वास्थ्य प्रदानकर्ता ने आपका वजन किया?** | No नहीं 0  Yes हां 1  Does not know पता नहीं.............................................................. 98 |
|  | 1. Were you weighed at more than one antenatal care visit?   **क्या आपको एक से ज़्यादा गर्भावस्था की जाँच के दौरान वज़न किया गया था?** | No नहीं 0  Yes हां 1  Does not know पता नहीं............................................................ 98 |
| a | What did you understand by ‘weigh you’?  ''वज़न किया'' - इससे आप क्या समझे? |  |
| b | How did you remember that a healthcare provider weighed you?  आपको कैसे याद है कि किसी स्वास्थ्य प्रदानकर्ता ने आपको वज़न किया था? |  |
| c | How do you remember that a heathcare provider weighed you more than once?  आपको ये कैसा याद है कि स्वस्थ्य प्रदानकर्ता ने आपको एक से अधिक बार वज़न किया था? |  |
| d | Who was the health care provider who weighed you?  आप को कौन से स्वस्थ्य प्रदानकर्ता ने वजन किया था? |  |
| e | Was this question easy or difficult for you to answer?  क्या आप को इस सवाल का जवाब देना आसान लगा की मुश्किल लगा? |  |
| f | Why did you feel this way?  आपको ऐसा क्यों लगा? |  |
| g | Do you think other women like you will find be able to respond to these questions? Or will they find it difficult?  क्या आपको लगता है की आप जैसी दूसरी महिलाऍं इस प्रश्न का उत्तर दे पायेंगी? या उनको मुश्किल लगेगा? |  |
| h | Why do you think so?  यदि मुश्किल लगा हो तो, ऐसा क्यों लगा था? |  |
|  | **Miscellaneous/ Additional Probes:** |  |
| **303** | As part of your antenatal care during this pregnancy, were any of the following done at least once:  **इस गर्भावस्था की जांच के दौरान क्या कम से कम एक बार** |  |
|  | 1. Did a health care provider talk with you about your weight?   **क्या किसी स्वास्थ्य प्रदानकर्ता ने आपके वज़न के बारे में आप से बात किया था?** | No नहीं 0  Yes हां 1  Does not know पता नहीं........................................................... 98 |
|  | 1. Did a health care provider talk with you about being physically active?   **क्या किसी स्वास्थ्य प्रदानकर्ता ने आपसे चलने फिरने या काम काज करने के बारे में बात की?** | No नहीं 0  Yes हां 1  Does not know पता नहीं 98 |
| a | Can you tell me in your own words what I have just asked you?  क्या आप मुझे अपने शब्दों में बता सकते है की मैंने आप से क्या पूछा? |  |
| b | What did the healthcare provider tell you about your weight?  आपको स्वास्थ्य प्रदानकर्ता ने वज़न के बारे में क्या बताया **था**? |  |
| c | Sometimes, it is difficult to remember what happened during pregnancy, how do you remember this?  आम तौर पर गर्भावस्था के दौरान हुई बातों को याद रखना मुश्किल होता है, आपको ये बातें कैसे याद है? |  |
| d | What did the healthcare provider tell you about being physically active?  स्वास्थ्य प्रदानकर्ता ने आपको गर्भावस्था के दौरान चलने फिरने के बारे में क्या बताया था? |  |
| e | How do you remember this?  आपको ये बात कैसे याद है? |  |
| f | Was this question easy or difficult for you to answer?  क्या आप को इस सवाल का जवाब देना आसान लगा की मुश्किल लगा? |  |
| g | Why did you feel this way?  आपको ऐसा क्यों लगा? |  |
| h | Do you think other women like you will find be able to respond to these questions? Or will they find it difficult?  क्या आपको लगता है की आप जैसी दूसरी महिलाऍं इस प्रश्न का उत्तर दे पायेंगी? या उनको मुश्किल लगेगा? |  |
| i | Why do you think so?  यदि मुश्किल लगा हो तो, ऐसा क्यों लगा था? |  |
|  | **Miscellaneous/ Additional Probes:** |  |
| **304** | During your pregnancy with *[NAME OF YOUNGEST CHILD*], did a health care provider or community health worker talk with you about taking iron tablets or iron syrup?  **जब (बच्चे का नाम) पेट में था, क्या किसी स्वास्थ्य प्रदानकर्ता या सामुदायिक स्वास्थ्य कार्यकर्ता ने आपके साथ आयरन की गोली या सिरप खाने के बारे में बात किया था?** | No नहीं 0  Yes हां 1  Does not know पता नहीं.......................................................... 98 |
| a. | Who talked with you about iron tablets?  **आयरन की गोली के बारे में किसने बताया था?** |  |
| b | What did the *[include from above response]* tell you about iron tablets or syrup?  आयरन की गोली **या सिरप** के बारे में ______________ ने आपसे क्या बात की थी? |  |
| c | According to you, who is a healthcare provider?  आपके हिसाब से स्वास्थ्य प्रदानकर्ता कौन है? |  |
| d | According to you, who is a community health worker?  आपके हिसाब से सामुदायिक स्वास्थ्य कार्यकर्ता कौन है? |  |
| e | Was this question easy or difficult for you to answer?  क्या आप को इस सवाल का जवाब देना आसान लगा की मुश्किल लगा? |  |
| f | Why did you feel this way?  आपको ऐसा क्यों लगा? |  |
| g | Do you think other women like you will find be able to respond to these questions? Or will they find it difficult?  क्या आपको लगता है की आप जैसी दूसरी महिलाऍं इस प्रश्न का उत्तर दे पायेंगी? या उनको मुश्किल लगेगा ? |  |
| h | Why do you think so?  यदि मुश्किल लगा हो तो, ऐसा क्यों लगा था? |  |
|  | **Miscellaneous/ Additional Probes:** |  |
| **305** | During your pregnancy with [*NAME OF YOUNGEST CHILD*], did a health care provider or community health worker talk with you about taking calcium tablets?  **जब (बच्चे का नाम) पेट में था, क्या किसी स्वास्थ्य प्रदानकर्ता या सामुदायिक स्वास्थ्य कार्यकर्ता ने आपके साथ कैल्शियम की गोली लेने के बारे में बात किया था?** | No नहीं 0  Yes हां 1  Does not know पता नहीं.......................................................... 98 |
| a. | Who talked with you about calcium tablets?  **कैल्शियम की गोली के बारे में किसने बताया था?** |  |
| b | What did the *[include from above response]* tell you about calcium tablets or syrup?  कैल्शियम की गोली या सिरप के बारे में ______________ ने आपसे क्या बात की थी? |  |
| c | What did the healthcare provider or community health worker tell you about calcium tablets?  कैल्शियम की गोली के बारे में स्वास्थ्य प्रदानकर्ता या सामुदायिक स्वास्थ्य कार्यकर्ता ने आपसे क्या बात की थी ? |  |
| d | Was this question easy or difficult for you to answer?  क्या आप को इस सवाल का जवाब देना आसान लगा की मुश्किल लगा? |  |
| e | Why did you feel this way?  आपको ऐसा क्यों लगा? |  |
| f | Do you think other women like you will find be able to respond to these questions? Or will they find it difficult?  क्या आपको लगता है की आप जैसी दूसरी महिलाऍं इस प्रश्न का उत्तर दे पायेंगी? या उनको मुश्किल लगेगा? |  |
| g | Why do you think so?  यदि मुश्किल लगा हो तो, ऐसा क्यों लगा था? |  |
|  | **Miscellaneous/ Additional Probes:** |  |
| **306** | When you were pregnant with [*NAME OF YOUNGEST CHILD*], did you receive any counseling about breastfeeding from any health care provider or community health worker?  **जब आप (बच्चे का नाम) से गर्भवती थे, तब क्या आपको किसी स्वास्थ्य प्रदानकर्ता या सामुदायिक स्वास्थ्य कार्यकर्ता से स्तनपान के बारे में सलाह मिला था?** | No नहीं 0  Yes हां 1  Does not know पता नहीं.......................................................... 98 |
| a | In your own words, can you tell me what I have just asked you?  क्या आप मुझे अपने शब्दों में बता सकते है कि मैंने आप से क्या पूछा? |  |
| b. | Who talked with you about breastfeeding?  **स्तनपान के बारे में किसने बताया था?** |  |
| c | What did the *[include from above response]* tell you about breastfeeding?  **स्तनपान के बारे** के बारे में ______________ ने आपसे क्या बात की थी? |  |
| d | Do you consider [*include from response to b]* a health care provider*?*  क्या आप इनको स्वास्थ्य प्रदानकर्ता मानते हैं? |  |
| e | Do you consider [*include from response to b]* a community health worker*?*  क्या आप इनको सामुदायिक स्वास्थ्य कार्यकर्ता मानते हैं? |  |
| f | When I asked you, during pregnancy if you received any counselling from a healthcare provider or community health worker about breastfeeding- what did you understand by “counsel about breastfeeding’’?  जब मैंने पूछा, क्या आपको गर्भावस्था के दौरान किसी स्वास्थ्य प्रदानकर्ता या सामुदायिक स्वास्थ्य कार्यकर्ता से स्तनपान के बारे में सलाह मिला - स्तनपान के बारे में सलाह से आप को क्या समझ में आया? |  |
| g | Was this question easy or difficult for you to answer?  क्या आप को इस सवाल का जवाब देना आसान लगा की मुश्किल लगा? |  |
| h | Why did you feel this way?  आपको ऐसा क्यों लगा? |  |
| i | Do you think other women like you will find be able to respond to these questions? Or will they find it difficult?  क्या आपको लगता है की आप जैसी दूसरी महिलाऍं इस प्रश्न का उत्तर दे पायेंगी? या उनको मुश्किल लगेगा? |  |
| j | Why do you think so?  यदि मुश्किल लगा हो तो, ऐसा क्यों लगा था? |  |
|  | **Miscellaneous/ Additional Probes:** |  |
| **307** | Immediately after birth, was [*NAME OF YOUNGEST CHILD*] put on your chest?  **जन्म के तुरंत बाद, क्या (बच्चे का नाम) को आपके छाती पर रखा गया था?** | No नहीं 0  Yes हां 1  Does not know पता नहीं.......................................................... 98 |
| a | According to you, what does “immediately after birth” mean?  आप के हिसाब से जन्म के तुरंत बाद का समय मतलब कौन सा समय होता है? |  |
| b | Some people find it difficult, how did you remember what happened immediately after birth?  आम तौर पर लोगों को मुश्किल हो सकता है\|, आपको कैसे याद है की जन्म के तुरंत बाद क्या हुआ था? |  |
| c | Was this question easy or difficult for you to answer?  क्या आप को इस सवाल का जवाब देना आसान लगा की मुश्किल लगा? |  |
| d | Why did you feel this way?  आपको ऐसा क्यों लगा? |  |
| e | Do you think other women like you will find be able to respond to these questions? Or will they find it difficult?  क्या आपको लगता है की आप जैसी दूसरी महिलाऍं इस प्रश्न का उत्तर दे पायेंगी? या उनको मुश्किल लगेगा? |  |
| f | Why do you think so?  यदि मुश्किल लगा हो तो, ऐसा क्यों लगा था? |  |
|  | **Miscellaneous/ Additional Probes:** |  |
| **308** | During the first two days after [*NAME OF YOUNGEST CHILD*]’s birth, did any health care provider or community health worker do the following:  (**बच्चे का नाम) के जन्म के दो दिनों के अंदर क्या किसी स्वास्थ्य प्रदानकर्ता या सामुदायिक स्वास्थ्य कार्यकर्ता ने** |  |
|  | 1. Talk with you about breastfeeding?   **स्तनपान के बारे में आपसे बात किया** | No नहीं 0  Yes हां 1  Does not know पता नहीं..........................................................98 |
| a | Usually two days after the the child’s birth is difficult, how do you remember this?  आम तौर पे बच्चे के जन्म के दो **दिनों के अंदर** का समय मुश्किल हो सकता है\| आप को उन दो दिनों में क्या हुआ था, यह कैसे याद है? |  |
| b. | Who talked with you about breastfeeding?  **स्तनपान के बारे में किसने बताया था?** |  |
| c | What did the *[include from above response]* tell you about breastfeeding?  **स्तनपान के बारे** के बारे में ______________ ने आपसे क्या बात की थी? |  |
| d | Was this question easy or difficult for you to answer?  क्या आप को इस सवाल का जवाब देना आसान लगा की मुश्किल लगा? |  |
| e | Why did you feel this way?  आपको ऐसा क्यों लगा? |  |
| f | Do you think other women like you will find be able to respond to these questions? Or will they find it difficult?  क्या आपको लगता है की आप जैसी दूसरी महिलाऍं इस प्रश्न का उत्तर दे पायेंगी? या उनको मुश्किल लगेगा? |  |
| g | Why do you think so?  यदि मुश्किल लगा हो तो, ऐसा क्यों लगा था? |  |
|  | **Miscellaneous/ Additional Probes:** |  |
| **308** | During the first two days after [*NAME OF YOUNGEST CHILD*]’s birth, did any health care provider or community health worker do the following    (**बच्चे का नाम) के जन्म के दो दिनों अंदर क्या किसी स्वास्थ्य प्रदानकर्ता या सामुदायिक स्वास्थ्य कार्यकर्ता ने** |  |
|  | 1. Observe [*NAME OF YOUNGEST CHILD*] breastfeeding?   **(बच्चे का नाम) को स्तनपान करते हुए देखा?** | No नहीं 0  Yes हां 1  Does not know पता नहीं 98 |
| a | Can you tell me what I have just spoken to you about?  क्या आप बता सकते हैं कि मैंने अभी किस बारे में बात किया? |  |
| b | Who observed you breastfeeding your child?  आप को अपने **बच्चे** को स्तनपान कराते हुए किसने देखा था? |  |
| c | How do you remember *[include from above response]* _________________ observed your child breastfeeding?  आपको कैसे याद है कि ___________________________ने आपको स्तनपान कराते हुए देखा था? |  |
| d | Was this question easy or difficult for you to answer?  क्या आप को इस सवाल का जवाब देना आसान लगा की मुश्किल लगा? |  |
| e | Why did you feel this way?  आपको ऐसा क्यों लगा? |  |
| f | Do you think other women like you will find be able to respond to these questions? Or will they find it difficult?  क्या आपको लगता है की आप जैसी दूसरी महिलाऍं इस प्रश्न का उत्तर दे पायेंगी? या उनको मुश्किल लगेगा? |  |
| g | Why do you think so?  यदि मुश्किल लगा हो तो, ऐसा क्यों लगा था? |  |
|  | **Miscellaneous/ Additional Probes:** |  |
| **INFANT AND YOUNG CHILD FEEDING COUNSELING** | | |
| **309** | In the last 6 months, did any health care provider or community health worker talk with you about how or what to feed your child?  **पिछले ६ महीनो में, क्या किसी स्वास्थ्य प्रदानकर्ता या सामुदायिक स्वास्थ्य कार्यकर्ता ने आपसे बच्चे को कैसे और क्या खिलाना चाहिए - इसके बारे में बात की?** | No नहीं 0  Yes हां 1  Does not know पता नहीं.......................................................... 98 |
| A | Can you tell me what I have just spoken to you about?  क्या आप बता सकते हैं कि मैंने अभी किस बारे में बात किया था? |  |
| B | When I said the last 6 months, then what time did you think of?  जब मैंने पिछले ६ महीने की बात की, तब आप को कौन सा समय याद आया था? |  |
| C | What do you understand by ‘how and what to feed your child’?  बच्चे को कैसे और क्या खिलाना चाहिए - इसके बारे में आप क्या समझते हैं? |  |
| d | Was this question easy or difficult for you to answer?  क्या आप को इस सवाल का जवाब देना आसान लगा की मुश्किल लगा? |  |
| e | Why did you feel this way?  आपको ऐसा क्यों लगा? |  |
| f | Do you think other women like you will find be able to respond to these questions? Or will they find it difficult?  क्या आपको लगता है की आप जैसी दूसरी महिलाऍं इस प्रश्न का उत्तर दे पायेंगी? या उनको मुश्किल लगेगा? |  |
| g | Why do you think so?  यदि मुश्किल लगा हो तो, ऐसा क्यों लगा था? |  |
|  | **Miscellaneous/ Additional Probes:** |  |
| **310** | In the last 6 months, what did the health care provider or community health worker talk with you regarding **how or what** to feed your child?  *Multiple responses possible.*  **पिछले ६ महीनो में, किसी स्वास्थ्य प्रदानकर्ता या सामुदायिक स्वास्थ्य कार्यकर्ता ने आपके साथ बच्चे को क्या और कैसे खिलाना चाहिए - इसके बारे में क्या बात की थी?** | Breastfeeding स्तनपान 1  Not feeding water or other liquids before six months, other than breastmilk……… 2  छह महीने पहले मां के दूध के अलावा पानी तथा अन्य लिक्विड ना पीलाना  Introducing food and liquids (other than breastmilk) when the baby reaches six months of age 3  शिशु छह महीने का होने के बाद खाने के पदार्थ और लिक्विड (मां के दूध के अलावा) देना शुरु करना  Giving a variety of foods विभिन्न खाद्यपदार्थ देना 4  Giving animal source foods (eggs, milk, meat, fish) 5  पशु स्त्रोत खाद्यपदार्थ (अंडे, दूध, मांस, मच्छी) देना  How often to feed foods 6  खाने के पदार्थ कितने समय के बाद खिलाना  Not feeding sugar-sweetened beverages 7  चीनीयुक्त मीठे पेय ना पीलाना  Not feeding unhealthy foods (sugary, salty or fried foods)  **स्वस्थ के लिए हानिकारक खाद्य पदार्थ (मीठा, नमकीन या तले खाद्य पदार्थ) न खिलाना**… 8  None of the above इनमें से कोई नहीं 9  Other (specify: ______) अन्य (बतायें) 95 |
| a | Can you tell me what I have just spoken to you about?  क्या आप बता सकते हैं कि मैंने अभी किस बारे में बात किया था? |  |
| b | When I said the last 6 months, what time did you think of?  जब मैंने पिछले ६ महीने की बात की तब आप को कौन सा समय याद आया था? |  |
| c | How do you remember what happened in the last 6 months?  आपको ये कैसा याद है कि पिछले ६ महीने में क्या हुआ था? |  |
| d | Was this question easy or difficult for you to answer?  क्या आप को इस सवाल का जवाब देना आसान लगा की मुश्किल लगा? |  |
| e | Why did you feel this way?  आपको ऐसा क्यों लगा? |  |
| f | Do you think other women like you will find be able to respond to these questions? Or will they find it difficult?  क्या आपको लगता है की आप जैसी दूसरी महिलाऍं इस प्रश्न का उत्तर दे पायेंगी? या उनको मुश्किल लगेगा? |  |
| g | Why do you think so?  यदि मुश्किल लगा हो तो, ऐसा क्यों लगा था? |  |
|  | **Miscellaneous/ Additional Probes:** |  |
| **311** | In the last 6 months, did any health care provider or community health worker talk with you about breastfeeding?  **पिछले ६ महीनो में, क्या किसी स्वास्थ्य प्रदानकर्ता या सामुदायिक स्वास्थ्य कार्यकर्ता ने आपके साथ स्तनपान के बारे में बात की?** | No नहीं 0  Yes हां 1  Does not know पता नहीं........................................................ 98 |
| a | What do you understand by breastfeeding?  आप **स्तनपान** से क्या समझते हैं? |  |
| b. | Who talked with you about breastfeeding in the last 6 months?  **पिछले ६ महीनो में, स्तनपान के बारे में किसने बताया था?** |  |
| c | What did the *[include from above response]* tell you about breastfeeding?  **स्तनपान के बारे** के बारे में ______________ ने आपसे क्या बात की थी? |  |
| d | Was this question easy or difficult for you to answer?  क्या आप को इस सवाल का जवाब देना आसान लगा की मुश्किल लगा? |  |
| e | Why did you feel this way?  आपको ऐसा क्यों लगा? |  |
| f | Do you think other women like you will find be able to respond to these questions? Or will they find it difficult?  क्या आपको लगता है की आप जैसी दूसरी महिलाऍं इस प्रश्न का उत्तर दे पायेंगी? या उनको मुश्किल लगेगा? |  |
| g | Why do you think so?  यदि मुश्किल लगा हो तो, ऐसा क्यों लगा था? |  |
|  | **Miscellaneous/ Additional Probes:** |  |
| **312** | In the last 6 months, did any health care provider or community health worker talk with you about giving your child liquids, semi-solid, or solid foods, other than breastmilk?  **पिछले ६ महीनो में, क्या किसी स्वास्थ्य प्रदानकर्ता या सामुदायिक स्वास्थ्य कार्यकर्ता ने आपके साथ अपने बच्चे को माँ के दूध के अलावा , तरल पदार्थ, अर्ध-ठोस (मसला हुआ) या ठोस खाद्य पदार्थ देने के बारे में बात किया?** | No नहीं 0  Yes हां 1  Does not know पता नहीं........................................................ 98 |
| a | Can you tell me what I have just spoken to you about?  क्या आप बता सकते हैं कि मैंने अभी किस बारे में बात किया ? |  |
| b | When I said the last 6 months, what time did you think of?  जब मैंने पिछले ६ महीने की बात की तब आप को कौन सा समय याद आया था? |  |
| c | What do you understand by liquid, semi solid and solid foods?  आप तरल पदार्थ, मसला हुआ/ अर्ध ठोस, या ठोस खाद्य पदार्थ से क्या समझते हैं? |  |
| d. | Who talked with you about liquid, semi solid and solid foods in the last 6 months?  **पिछले ६ महीनो में,** तरल पदार्थ, मसला हुआ/ अर्ध ठोस, या ठोस खाद्य पदार्थ **के बारे में किसने बताया था?** |  |
| e | What did the *[include from above response]* tell you about liquid, semi solid and solid foods?  तरल पदार्थ, मसला हुआ/ अर्ध ठोस, या ठोस खाद्य पदार्थ **के बारे** के बारे में ______________ ने आपसे क्या बात की थी? |  |
| f | Was this question easy or difficult for you to answer?  क्या आप को इस सवाल का जवाब देना आसान लगा की मुश्किल लगा? |  |
| g | Why did you feel this way?  आपको ऐसा क्यों लगा? |  |
| h | Do you think other women like you will find be able to respond to these questions? Or will they find it difficult?  क्या आपको लगता है की आप जैसी दूसरी महिलाऍं इस प्रश्न का उत्तर दे पायेंगी? या उनको मुश्किल लगेगा? |  |
| i | Why do you think so?  यदि मुश्किल लगा हो तो, ऐसा क्यों लगा था? |  |
|  | **Miscellaneous/ Additional Probes:** |  |
| **313** | In the last 6 months, did a health care provider or community health worker talk with you about:  **पिछले ६ महीनो में क्या किसी स्वास्थ्य प्रदानकर्ता या सामुदायिक स्वास्थ्य कार्यकर्ता ने** |  |
|  | 1. Not feeding water or other liquids before 6 months, other than breastmilk   **६ महीने के पहले माँ के दूध के अलावा, पानी या किसी और तरल पदार्थ को न देने के बारे में बात की** | No नहीं 0  Yes हां 1  Does not know पता नहीं........................................................ 98 |
| a | Can you tell me what I have just spoken to you about?  क्या आप बता सकते हैं कि मैंने अभी किस बारे में बात किया था? |  |
| b | Was this question easy or difficult for you to answer?  क्या आप को इस सवाल का जवाब देना आसान लगा की मुश्किल लगा? |  |
| c | Why did you feel this way?  आपको ऐसा क्यों लगा? |  |
| d | Do you think other women like you will find be able to respond to these questions? Or will they find it difficult?  क्या आपको लगता है की आप जैसी दूसरी महिलाऍं इस प्रश्न का उत्तर दे पायेंगी? या उनको मुश्किल लगेगा? |  |
| e | Why do you think so?  यदि मुश्किल लगा हो तो, ऐसा क्यों लगा था? |  |
|  | **Miscellaneous/ Additional Probes:** |  |
| **313** | In the last 6 months, did a health care provider or community health worker talk with you about:  **पिछले ६ महीनो में क्या किसी स्वास्थ्य प्रदानकर्ता या सामुदायिक स्वास्थ्य कार्यकर्ता ने** |  |
|  | 1. Introducing food and liquids (other than breastmilk) when the baby reaches 6 months of age   **६ महीनो के पूरे होने पर, माँ के दूध के अलावा बच्चे को खाद्य और तरल पदार्थो को देने के बारे में बात की** | No नहीं 0  Yes हां 1  Does not know पता नहीं........................................................ 98 |
| a | Can you tell me what I have just spoken to you about?  क्या आप बता सकते हैं कि मैंने अभी किस बारे में बात किया था? |  |
| b | Who talked with you about introducing food and liquids (other than breastmilk) when the baby reaches 6 months of age?  **पिछले ६ महीनो में,** तरल पदार्थ, मसला हुआ/ अर्ध ठोस, या ठोस खाद्य पदार्थ **के बारे में आप को किसने बताया था?** |  |
| c | Was this question easy or difficult for you to answer?  क्या आप को इस सवाल का जवाब देना आसान लगा की मुश्किल लगा? |  |
| d | Why did you feel this way?  आपको ऐसा क्यों लगा? |  |
| e | Do you think other women like you will find be able to respond to these questions? Or will they find it difficult?  क्या आपको लगता है की आप जैसी दूसरी महिलाऍं इस प्रश्न का उत्तर दे पायेंगी? या उनको मुश्किल लगेगा? |  |
| f | Why do you think so?  यदि मुश्किल लगा हो तो, ऐसा क्यों लगा था? |  |
|  | **Miscellaneous/ Additional Probes:** |  |
| **313** | In the last 6 months, did a health care provider or community health worker talk with you about:  **पिछले ६ महीनो में क्या किसी स्वास्थ्य प्रदानकर्ता या सामुदायिक स्वास्थ्य कार्यकर्ता ने** |  |
|  | 1. Giving a variety of foods   **आपके बच्चे को तरह तरह की खाद्य पदार्थ देने के बारे में बात की** | No नहीं 0  Yes हां 1  Does not know पता नहीं........................................................ 98 |
| a | What do you understand by a variety of foods?  आप तरह तरह के खाद्य पदार्थ से क्या समझते हैं? |  |
| b | Was this question easy or difficult for you to answer?  क्या आप को इस सवाल का जवाब देना आसान लगा की मुश्किल लगा? |  |
| c | Why did you feel this way?  आपको ऐसा क्यों लगा? |  |
| d | Do you think other women like you will find be able to respond to these questions? Or will they find it difficult?  क्या आपको लगता है की आप जैसी दूसरी महिलाऍं इस प्रश्न का उत्तर दे पायेंगी? या उनको मुश्किल लगेगा? |  |
| e | Why do you think so?  यदि मुश्किल लगा हो तो, ऐसा क्यों लगा था? |  |
|  | **Miscellaneous/ Additional Probes:** |  |
| **313** | In the last 6 months, did a health care provider or community health worker talk with you about:  **पिछले ६ महीनो में क्या किसी स्वास्थ्य प्रदानकर्ता या सामुदायिक स्वास्थ्य कार्यकर्ता ने** |  |
|  | 1. Giving animal source foods (such as eggs, milk, meat or fish)   **अंडे, दूध, मांस या मछली देना के बारे में बात की** | No नहीं 0  Yes हां 1  Does not know पता नहीं........................................................ 98 |
| a | In the past 6 months, who talked with you about giving eggs, milk, meat or fish?  **पिछले ६ महीनो में, अंडे, दूध, मांस या मछली देना के बारे में आप से किसने बात की?** |  |
| b | Was this question easy or difficult for you to answer?  क्या आप को इस सवाल का जवाब देना आसान लगा की मुश्किल लगा? |  |
| c | Why did you feel this way?  आपको ऐसा क्यों लगा? |  |
| d | Do you think other women like you will find be able to respond to these questions? Or will they find it difficult?  क्या आपको लगता है की आप जैसी दूसरी महिलाऍं इस प्रश्न का उत्तर दे पायेंगी? या उनको मुश्किल लगेगा? |  |
| e | Why do you think so?  यदि मुश्किल लगा हो तो, ऐसा क्यों लगा था? |  |
|  | **Miscellaneous/ Additional Probes:** |  |
| **313** | In the last 6 months, did a health care provider or community health worker talk with you about:  **पिछले ६ महीनो में क्या किसी स्वास्थ्य प्रदानकर्ता या सामुदायिक स्वास्थ्य कार्यकर्ता ने** |  |
|  | 1. Not feeding sugar-sweetened beverages   **आपके बच्चे को चीनी डाला हुआ मीठा पेय को न खिलाने के बारे में बात की** | No नहीं 0  Yes हां 1  Does not know पता नहीं 98 |
|  | 1. Not feeding sugary, salty or fried foods   **चीनी डाला हुआ मीठा, नमकीन या तले हुए खाद्य पदार्थ नहीं खिलाने के बारे में?** | No नहीं 0  Yes हां 1  Does not know पता नहीं 98 |
| a | According to you, what are sugar sweetened beverages?  आपके हिसाब से चीनी डाला हुआ मीठा पेय क्या होता है? |  |
| b | According to you, what are sugary, salty and fried foods?  आपके हिसाब से चीनी डाला हुआ मीठा, नमकीन या तले हुए खाद्य पदार्थ क्या होता है? |  |
| c | According to you, what are unhealthy foods?  आपके हिसाब से "स्वस्थ के लिए हानिकारक खाद्य पदार्थ" क्या हैं? |  |
| d | Was this question easy or difficult for you to answer?  क्या आप को इस सवाल का जवाब देना आसान लगा की मुश्किल लगा? |  |
| e | Why did you feel this way?  आपको ऐसा क्यों लगा? |  |
| f | Do you think other women like you will find be able to respond to these questions? Or will they find it difficult?  क्या आपको लगता है की आप जैसी दूसरी महिलाऍं इस प्रश्न का उत्तर दे पायेंगी? या उनको मुश्किल लगेगा? |  |
| g | Why do you think so?  यदि मुश्किल लगा हो तो, ऐसा क्यों लगा था? |  |
|  | **Miscellaneous/ Additional Probes:** |  |
| **314** | In the last 6 months, where did the health care provider or community health worker talk with you about how or what to feed your child?  *Multiple responses possible.*  **पिछले 6 महीनों में, स्वास्थ्य प्रदानकर्ता या सामुदायिक स्वास्थ्य कार्यकर्ता ने आपसे कहाँ पर बच्चे को कैसे और क्या खिलाना चाहिए के बारे में बात की थी?** | At home घर में 1  At the Anganwadi center आंगनवाड़ी केंद्र में 2  At health center स्वास्थ्य केंद्र में 3  Hospital अस्पताल 4  Private clinic निजी दवाखाना 5  Outreach facility आउटरीच सुविधा 6  NGO facility एनजीओ सुविधा 7  In the community/village समुदाय / गांव में 8  Other (specify: ______) अन्य (बतायें) 95 |
| a | Can you tell me what I have just spoken to you about?  क्या आप बता सकते हैं की मैंने अभी क्या पूछा? |  |
| b | How do you remember this/ these place(s)?  आपको इस/ इन जगहों के बारे में कैसा याद है? |  |
| c | Was this question easy or difficult for you to answer?  क्या आप को इस सवाल का जवाब देना आसान लगा की मुश्किल लगा? |  |
| d | Why did you feel this way?  आपको ऐसा क्यों लगा? |  |
| e | Do you think other women like you will find be able to respond to these questions? Or will they find it difficult?  क्या आपको लगता है की आप जैसी दूसरी महिलाऍं इस प्रश्न का उत्तर दे पायेंगी? या उनको मुश्किल लगेगा? |  |
| f | Why do you think so?  यदि मुश्किल लगा हो तो, ऐसा क्यों लगा था? |  |
|  | **Miscellaneous/ Additional Probes:** |  |
| **315** | In the last 6 months, which health worker talked with you about how or what to feed your child?  *Multiple responses possible.*  **पिछले 6 महीनों में, कौन से स्वास्थ्य कार्यकर्ता ने आपसे बच्चे को कैसे और क्या खिलाना चाहिए के बारे में बात की थी?** | Anganwadi Worker **आंगनवाड़ी कार्यकर्ता** 1  ASHA आशा 2  ANM एएनएम 3  Doctor डॉक्टर 4  Pharmacist फार्मासिस्ट 5  NGO worker एनजीओ कर्मचारी 6  Other (specify: ______) अन्य (बतायें) 95  Does not know पता नहीं 98 |
| a | How do you remember this/these health worker(s)?  आपको ये / इन स्वास्थ्य कार्यकर्ता कैसे याद है? |  |
| b | Was this question easy or difficult for you to answer?  क्या आप को इस सवाल का जवाब देना आसान लगा की मुश्किल लगा? |  |
| c | Why did you feel this way?  आपको ऐसा क्यों लगा? |  |
| d | Do you think other women like you will find be able to respond to these questions? Or will they find it difficult?  क्या आपको लगता है की आप जैसी दूसरी महिलाऍं इस प्रश्न का उत्तर दे पायेंगी? या उनको मुश्किल लगेगा? |  |
| e | Why do you think so?  यदि मुश्किल लगा हो तो, ऐसा क्यों लगा था? |  |
|  | **Miscellaneous/ Additional Probes:** |  |
